# Answer to Inquirer

**Published:** 1888-02

**Authors:** 


ANSWER TO INQUIRER.—Two answers were received to *Inquirer*, in our last issue; one from New York, the other from Philadelphia. Grindelia rob. was recommended for the case, by both physicians.

